# Times series analysis of age-specific tuberculosis at a rapid developing region in China, 2011–2016

**DOI:** 10.1038/s41598-018-27024-w

**Published:** 2018-06-07

**Authors:** Minmin Zhu, Guiyuan Han, Howard Eugene Takiff, Jian Wang, Jianping Ma, Min Zhang, Shengyuan Liu

**Affiliations:** 1 Shenzhen Nanshan Center for Chronic Disease Control, Shenzhen, 518054 China; 2 Institut Pasteur, Unité de Génétique Mycobacterienne, Paris, 75015 France; 3 Instituto Venezolano de Investigaciones Cientificas, Caracas, Venezuela

## Abstract

The city of Shenzhen has recently experienced extraordinary economic growth accompanied by a huge internal migrant influx. We investigated the local dynamics of tuberculosis (TB) epidemiology in the Nanshan District of Shenzhen to provide insights for TB control strategies for this district and other rapidly developing regions in China. We analyzed the age-specific incidence and number of TB cases in the Nanshan District from 2011 to 2016. Over all, the age-standardized incidence of TB decreased at an annual rate of 3.4%. The incidence was lowest amongst the age group 0–14 and showed no increase in this group over the six-year period (*P* = 0.587). The fastest decreasing incidence was among the 15–24 age group, with a yearly decrease of 13.3% (*β* = 0.867, *P* < 0.001). In contrast, the TB incidence increased in the age groups 45–54, 55–54, and especially in those aged ≥65, whose yearly increase was 13.1% (*β* = 1.131, *P* < 0.001). The peak time of TB case presentation was in April, May, and June for all age groups, except in August for the 45–54 cohort. In the rapidly developing Nanshan District, TB control policies targeted to those aged 45 years and older should be considered. The presentation of TB cases appears to peak in the spring months.

## Introduction

Despite its remarkable economic growth, China continues to be one of the high-burden countries for tuberculosis (TB), according to the global TB report 2017^1^. The ambitious targets in the “End TB Strategy” proposed by the World Health Organization in 2015 pose a real challenge for TB control, and considerable work remain to be done in China^2^.

China has the world’s largest internal migrant population, which brings great impacts on the public health^3,4^. In China, nearly 10% of TB patients were diagnosed and treated in cities different from their official residence address, and they mainly flowed from the central or western provinces to the eastern provinces^5^. In Shenzhen, which is at the forefront of China’s economic reform and opening-up, this floating population has an even greater impact on the local TB epidemiology. From 2001 to 2010, registered pulmonary TB (PTB) cases among migrants accounted for nearly 80% of the total PTB cases in Shenzhen, and the decline in the incidence of PTB among migrants was much slower than the decline amongst long-term local residents (70.6% VS 27.2%)^6^. In addition, non-adherence to anti-TB drug regimens was more common amongst the migrant TB patients^7^.

At the end of 2011, Shenzhen began to implement an industry upgrading policy to attract more high-tech companies, which was accompanied by a gradual change in the demographic characteristics of the city. From 2010 to 2015, the proportion of the population aged 0–14 years and aged ≥65 increased, and the prevalence of residents with a college education increased from 17644 to 22668 per 100000 population^8^.

TB incidence may be affected by demographic factors, such as age and gender. Worldwide, there are more male cases than female cases, with an estimated sex ratio of 1.7:1^1^, and even higher ratios in Asia-Pacific region^9^. Most TB cases occur in the population that is 55 years or older in Western Pacific^10^. Recently there has been more attention given to cases in children, who account for 10–20% of the disease burden in TB-endemic areas^11^.

The aims of this study were to investigate the age-specific temporal variations and peak time of TB case presentation in the Nanshan District of Shenzhen, within the context of industry upgrading and changing demographics. This study could provide important insights for TB control strategies in Nanshan, which would also benefit other Chinese cities that are similarly experiencing rapid economic development.

In Nanshan, the overall unadjusted incidence of TB has witnessed a continuous decline, with an annual decrease of 6% between 2011 and 2015. For subareas of Nanshan, a spatial-temporal analysis study revealed a low-but-rising TB incidence in the district’s central region, where high tech enterprises were clustered, and a high-but-declining incidence of TB in the northwest region where several labor-intensive factories are located^12^. However, the associations between demographics and TB incidence have not been analyzed.

Poisson regression based Serfling methods have proved accurate for estimation of seasonal variation in the presentation of cases and have also been used to detect TB outbreaks^13,14^. We similarly employed a Poisson Serfling regression model to estimate the seasonal amplitude in TB cases of Nanshan.

## Results

### General characteristics of TB cases

From 2011 to 2016, there were a total of 5497 TB cases reported in Nanshan, whose general characteristics are shown in Table 1. The two age groups with the greatest percentage of the TB cases were 25–34 years and 15–24 years, accounting for 36.9% and 29.0% of total TB cases, respectively. There were nearly twice as many cases in males as in females. About 44.8% of PTB cases were clinically diagnosed without microbiological confirmation. Only 7 extra-PTB cases were reported in the years 2014–2016, but in China pleural disease is registered as PTB. Using the WHO criteria of extra-pulmonary TB, which includes pleural tuberculosis involvement, there were 575 extra-pulmonary TB cases in Nanshan, which accounted for 10.5% of all TB patients, a little more than the average 8% in China, according to the global tuberculosis report 2017^1^.

**Table 1:** Characteristics of TB cases at Nanshan District, Shenzhen City, 2011–2016.

	2011	2012	2013	2014	2015	2016	total	Proportion (%)
All TB	1095	915	866	867	939	815	5497	100
**Age**, **years**								
0–14	2	0	3	2	2	3	12	0.2
15–24	345	308	264	246	236	196	1595	29.0
25–34	391	350	324	300	365	298	2028	36.9
35–44	174	140	131	128	136	114	823	15.0
45–54	85	58	71	98	101	92	505	9.2
55–64	57	39	49	50	53	58	306	5.6
≥65	41	20	24	43	46	54	228	4.1
**Gender**								
Female	370	338	299	282	334	274	1897	34.5
Male	725	577	567	585	605	541	3600	65.5
**TB diagnosis**								
**PTB**								
SS−	519	375	355	390	431	392	2462	44.8
SS+	373	318	314	286	325	213	1829	33.3
Culture+	86	107	103	105	109	121	631	11.5
pleurisy	117	115	94	85	69	88	568	10.3
**Extra-PTB**	0	0	0	1	5	1	7	0.1

Notes: ≥65:65 years and older; PTB: pulmonary tuberculosis; SS−: sputum smear negative; SS+: sputum smear positive; Culture+: sputum culture positive only; pleurisy: tuberculous pleurisy; Extra-PTB: extra-pulmonary tuberculosis.

### Annual notified TB incidence

**Figure 1:**
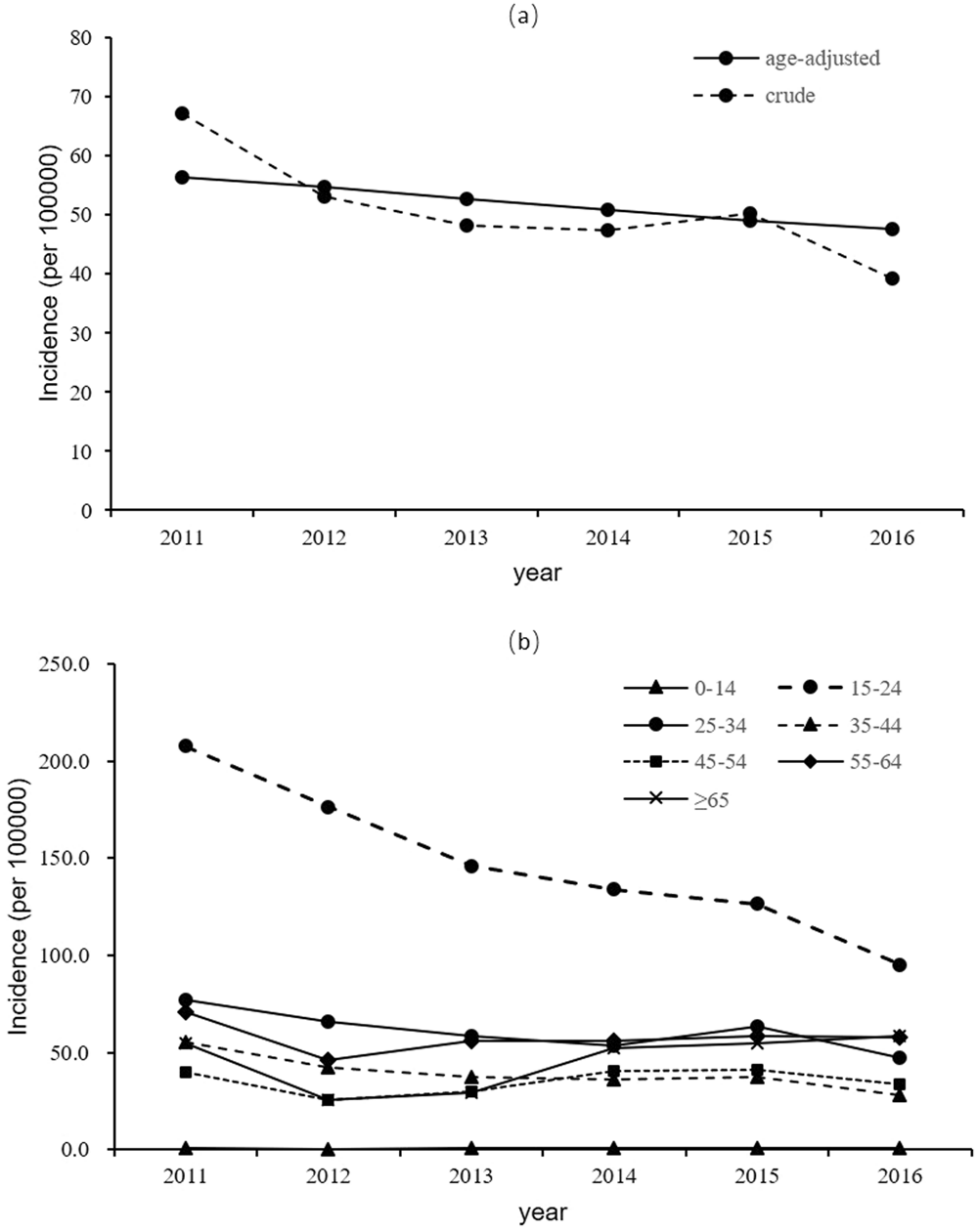
Yearly notified TB incidence in Nanshan. (**a**) Crude incidence among whole population and age-adjusted incidence using the WHO standard World population age distribution; (**b**) age-specific crude incidence.

The crude incidence of TB for the entire population of Nanshan decreased from 67.2 per 100000 population in 2011 to 48.3 in 2013, followed by a smaller decrease from 2013 to 2015, and then a marked decrease in 2016. After adjusting for age, however, the trend showed a less steep decline. The adjusted incidence for the entire population dropped from 56.4 in 2011 to 47.5 in 2016, with an annual decline rate of 3.4% (*P* < 0.001) (see Fig. 1a).

The age-specific TB incidence is illustrated in Table 2 and Fig. 1b. From 2011 to 2016, the incidence amongst the age group 0–14 years remained lower than 1 per 100000 population and did not change significantly over the six-year period (*P* = 0.587). After adjusting for gender and population size, the TB incidence showed a significant decrease in the age groups 15–24, 25–34 and 35–44, while increasing in the older age-groups 45–54, 55–64, ≥65. Although the crude TB incidence was consistently highest amongst those aged 15–24 years, with an average incidence of 145.3 during the study period, the incidence dropped from 207.6 in 2011 to 95.2 in 2016, with a yearly decrease of 13.3% (*β* = 0.867, *P* < 0.001). In contrast, the incidence among those aged ≥65 shifted from the 5^th^ highest incidence (54.9) age group in 2011 to the 2^nd^ highest (58.5) in 2016, with an annual increase of 13.1% (*β* = 1.131, *P* < 0.001).

**Table 2:** Secular trend in Poisson regression models for age-specific yearly TB incidence.

age group	*β*	95% *CI* of *β*	*P* value	
Age 0–14	1.098	0.784–1.536	0.587	
Age 15–24	0.867	0.843–0.893	<0.001	^***^
Age 25–34	0.963	0.956–0.970	<0.001	^***^
Age 35–44	0.936	0.925–0.947	<0.001	^***^
Age 45–54	1.067	1.051–1.083	<0.001	^***^
Age 55–64	1.027	1.007–1.048	0.008	^**^
Age ≥ 65	1.131	1.105–1.158	<0.001	^***^

Notes: The dependence variation of the model was the logarithm of yearly TB case number, independence was year, offset was log(population), and adjusted by gender; ≥65: 65 years and older; ^***^<0.001, ^**^<0.01, ^*^<0.05; *β* was equal to exp(coefficient) of the model.

### Monthly TB cases’ trend and peak time detection

**Figure 2:**
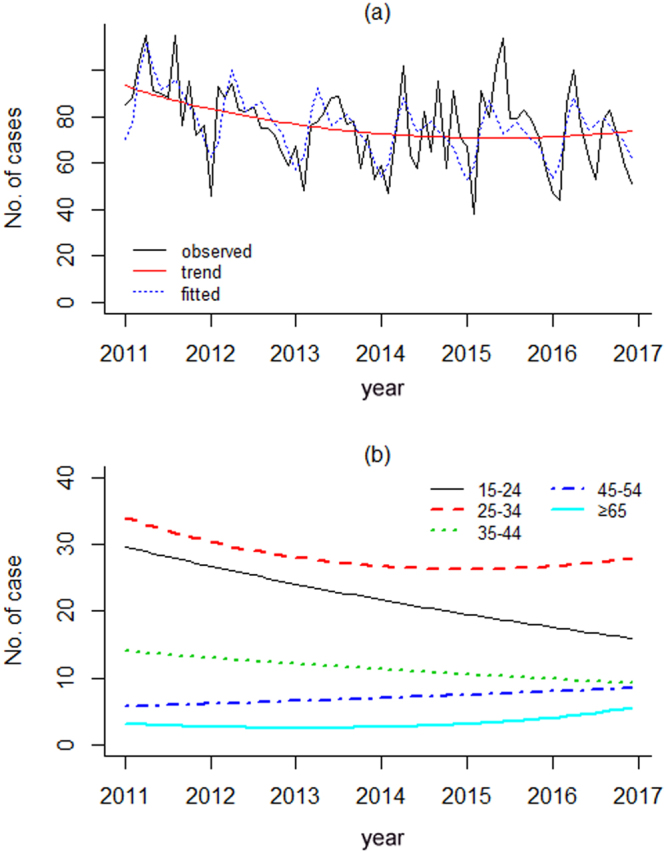
Monthly reported TB cases in Nanshan with secular trend and fitted value by Serfling regression model. (**a**) Observed TB cases in whole population, secular trend and fitted value; (**b**) age-spec1ific secular trends.

Using more detailed monthly data, a Serfling regression model showed a rising quadratic trend for all TB cases. Very similar to the yearly incidence data, increasing trends were detected among the age-groups of 45–54 and ≥65, and decreasing trends amongst age-groups 15–24, 25–34 and 35–44 (see Fig. 2).

The peak months for the presentation of TB cases was May for age-groups of 15–24 and 55–64, June for those aged 25–34 and 35–44, August for the age group 45–54, and April for age-group of ≥65. The amplitudes were less than 20% for those aged 25–34, 34–44 and 45–54 years old, and more than 40% for age groups of 55–64 and ≥65 (see Table 3).

**Table 3:** Age-specific TB peak time detected by Serfling regression models in Nanshan.

age group	Amplitude (%)	RR	RD	Peak month
All ages	24.1	1.6	0.5	May
Age 0–14*	—	—	—	—
Age 15–24	34.8	2.1	0.7	May
Age 25–34	15.5	1.5	0.4	June
Age 35–44	9.1	1.2	0.2	June
Age 45–54	12.1	1.3	0.2	August
Age 55–64	53.5	2.1	0.5	May
Age ≥ 65	45.5	2.0	0.7	April

Notes: RR: Related Risk = Peak/Trough, RD: Risk Difference = Peak-Trough; ≥ 65: 65 years and older; ^*^Age group of 0–14 was showed in randomized distribution without any long and season trend.

## Discussion

We used age-specific TB data and regression models to estimate the temporal changes in incidences and the peak time of case presentation among different age groups in Nanshan in the period from 2011 to 2016. We found that the variations of the temporal changes and peak time among different age groups pose challenges to TB control.

The TB incidence in Nanshan showed a decreasing trend in the 25–34 and 35–44 age groups, with the greatest annual decrease (13.3%) in the 15–24 group. We also found that the incidence of TB in the 0–14 age group, which makes up an increasing proportion of the population, was consistently the lowest of all age groups and shows no evidence of increasing. This is distinct from the situation in most of China where, from 2010 to 2015, the incidence of TB increased among children both under 5 (10.68 to 16.59) and 5–14 years (15.62 to 21.32)^15^.

Children and adolescents are generally at the highest risk for TB infection^16,17^, so the decreasing incidence among the younger population could indicate a decrease in active TB transmission in Nanshan as a result of the implementation of local TB control measures.

Contrary to the stable decreases among other age groups, during the 2011 to 2016 period we detected an increase in the TB incidence among age groups 45–54, 55–64 and ≥65. It appears plausible that the group of ≥65 could soon replace 15–24 as the age group with the highest incidence of TB. Similar results were found in a study conducted in Guangzhou, another city experiencing rapid economic development where the majority of the population are migrants. In that study of 136394 confirmed TB cases from 2007 to 2012, there was an increasing TB incidence in patients identified as the floating or migrant population and those aged 45–64^18^.

In the background of an ageing population in China and a maturing demographic in Shenzhen City and Nanshan District, TB control policies targeted to the population of age 45 and older should be considered. A mathematical model of TB transmission at the country level in China suggested that a combination of active case finding among the >65 population, increasing DOTS coverage, reducing treatment time, and increasing treatment success rates could reduce TB incidence by 59% (50–76%), and with the addition of preventative therapy for the elderly the incidence could be reduced by 84% (78–93%)^19^.

Studies of the seasonality of TB reported in the United States^20^, Israel^21^, India^22^, Portugal^23^ and China^24–27^, have shown that the spring months are often the peak period of TB case presentation, although some studies found that the seasonal trend varied with age. Kohei *et al*. analyzed the active TB cases in Japan from 1998 to 2013 using maximum entropy spectral analysis and the least squares method^28^. They observed that the peak time was June and July for the 10–39 age group, while the peak for the ≥70 age group was in August and September. A study on active tuberculosis cases in the mainland of China from 2005 to 2012 showed that May was the peak presentation month for the age group of 0–14, April for age groups of 15–24 and ≥65, and March for those aged 25–44 and 45–64^27^. In our study, the predominant peak time for most age groups was during the spring months (April, May or June), except for the age-group of 45–54, whose incidence peaked in August.

The mechanisms responsible for the seasonality of tuberculosis are not clear. Some studies have shown that TB seasonality correlated with the concentration of PM2.5 particulate matter in the air^29^ and the latitude^30^. Others have proposed that the increased transmission due indoor crowding and vitamin D deficiency during the winter followed by an incubation period and then delays in the diagnosis could potentially explain a spring peak for TB incidence^31,32^. In Shenzhen, the mean delays for care seeking and diagnosis among all TB patients were 34.0 and 15.1 days, respectively^33^. However, the longest delay in care seeking was 50.5 days among the age group of 45–60, which might partially explain the later August presentation peak for the age group of 45–54.

There are potential limitations in this study. First, there is no information on the official registered household address of the patients in the data, so it was not possible to determine what percentage of each age cohort of TB patients were migrants. The TB referral and diagnostic procedures in Nanshan are the same for both residents and migrants. Second, it is possible that there was an underreporting of TB cases in one or more age groups that would alter the observed trends. This seems unlikely, however, because in the WHO TB report 2017, China was estimated to have a low level of underreporting. In addition, in Nanshan the TB examinations and drugs are free, and patients receive reimbursements for transportation and nutrition.

## Conclusion

In Nanshan, within the context of industry upgrading and a rapidly developing economy, the instituted TB control activities including active case finding, high treatment success and strict school TB control, appear to have been successful in reducing the disease incidence in the younger population. TB control policies targeted to the migrating and resident populations aged 45 years and older should also be considered. The presentation of TB cases appears to peak in the spring months, except for the summer peak among the age group of 45–54.

## Methods

### Setting

The Nanshan district is in the southwest of the city of Shenzhen, across the bay from Hong Kong, with the geographic coordinates of latitude from 22°24′ to 22°39′ and of longitude from 113°53′ to 114°1′. Nanshan has a population of 1.36 million, of whom 0.55 million (40.3%) are migrants. Its GDP ranked 3^rd^ of all counties in China^34^, with a per capita GDP that reached US$43,700 in 2016. There were more than 2000 high-tech enterprises and 122 listed companies in Nanshan, ranking 2^nd^ of all counties in China.

Besides the fast economic development, Nanshan has also been a pilot site for introducing new TB control strategies in China. In 1995, Nanshan was one of the first counties in China to launch the DOTS (Directly Observed Therapy, Short-course) strategy, supported by a TB Control project funded by the World Bank. In 2006, Nanshan initiated the “*Floating Population Tuberculosis Control project*” and “*MDR Tuberculosis Control project*”, which were sponsored by the Fifth Round of the China - Global Fund TB Program, resulting in a good relationship between the floating TB patients and public health institutions^35^. Also, in Nanshan a three-tier TB control network was established, stipulating that all the community health centers and hospitals must report and refer the suspicious TB cases to a single government TB health center. Perhaps partially owing to free anti-TB medication and medical tests, in addition to transportation-and-nutrition reimbursement and rigorous DOTS, the treatment success rate for new smear positive TB patients is greater than 85%^6^. Based on National Operation Specification of School TB Control (pilot edition in 2010 and updated edition in 2017), Nanshan developed a local handbook for TB control in schools in 2017. The measures outlined include daily school TB surveillance, health education, preventing TB patients from attending school until they are successfully treated, and strict investigation of close contacts.

### Data

We retrospectively analyzed all TB cases newly reported to the National Information System for Disease Control and Prevention (NISDCP) from 2011 to 2016, according to the audit date of each case report. Both pulmonary and extra-pulmonary cases were included in the study. PTB cases were further categorized into four groups: sputum smear positive (SS+), sputum smear negative but culture positive (Culture+), clinically diagnosed cases (SS−), and tuberculosis pleurisy (Pleurisy). Pleural disease, either with or without TB disease in the lungs, is reported as PTB, according to the criteria in China. The population data were obtained from the local government of the Nanshan District of Shenzhen.

As all data on TB patients in this study were collected retrospectively from the NISDCP, patient informed consent was not required. The study was approved by the Institutional Review Board of the Shenzhen Nanshan Center for Chronic Disease Control. The datasets analyzed during the current study are available from the corresponding author upon reasonable request.

### Statistical Analysis

The yearly crude age-specific incidence was calculated as age-specific cases divided by the mid-year population. The WHO standard world population was used to generate age-adjusted incidence by direct standardization^36^.

Secular trends of age-specific TB incidence were analyzed by multivariate Poisson regressions, using the general linear model with log link and adjusted by variation of gender. In accordance with the Poisson model (count model), the log (incidence) was decomposed into log(cases) and log(population), with the latter being moved to the right of the equation. The multivariate Poisson regression model for each age group was:

1 $${\rm{l}}{\rm{o}}{\rm{g}}({\rm{c}}{\rm{a}}{\rm{s}}{\rm{e}}{\rm{s}})={\rm{o}}{\rm{f}}{\rm{f}}{\rm{s}}{\rm{e}}{\rm{t}}({\rm{l}}{\rm{o}}{\rm{g}}({\rm{p}}{\rm{o}}{\rm{p}}{\rm{u}}{\rm{l}}{\rm{a}}{\rm{t}}{\rm{i}}{\rm{o}}{\rm{n}}))+{\alpha }_{0}+{\alpha }_{1}\ast {\rm{y}}{\rm{e}}{\rm{a}}{\rm{r}}+{\alpha }_{2}\ast {\rm{g}}{\rm{e}}{\rm{n}}{\rm{d}}{\rm{e}}{\rm{r}}+\varepsilon ,$$

where “year” was the year when cases were reported and audited, “gender” was dummied as “female = 0, male = 1”, and “ε” was the random residual.

The Poisson Serfling cyclical regression method using monthly TB cases was performed to analyze the temporal trends and to detect the peak months of TB case presentation for each age group. The coefficients of the regression model were estimated by ordinary least squares, and model selection was also based on minimization of the Akaike information criterion (AIC). For each age-group, the full Poisson Serfling cyclical regression model was:

2 $$\begin{array}{c}\mathrm{Log}({{\rm{Y}}}_{{\rm{t}}})={{\rm{\alpha }}}_{0}+{{\rm{\alpha }}}_{1}{\rm{t}}+{{\rm{\alpha }}}_{2}{{\rm{t}}}^{2}+{{\rm{\gamma }}}_{1}\ast \cos (2{\rm{\pi }}t/12)+{{\rm{\delta }}}_{1}\ast \sin (2{\rm{\pi }}t/12)+{{\rm{\gamma }}}_{2}\ast \cos (4{\rm{\pi }}t/12)\\ \,\,\,\,\,+{{\rm{\delta }}}_{2}\ast \sin (4{\rm{\pi }}t/12)+{{\rm{\gamma }}}_{3}\ast \cos (6{\rm{\pi }}t/12)+{{\rm{\delta }}}_{3}\ast \sin (6{\rm{\pi }}t/12)\\ \,\,\,\,\,+{{\rm{\gamma }}}_{4}\ast \cos (8{\rm{\pi }}t/12)+{{\rm{\delta }}}_{4}\ast \sin (8{\rm{\pi }}t/12)+{{\rm{\varepsilon }}}_{{\rm{t}}},\end{array}$$

where Y_t_ is the monthly reported TB cases; t is the month of the TB cases reported, and t = 1, 2, 3,…, 72. In the model, there are secular trends and cyclical components for seasonal trends.

All the data were processed by Microsoft Excel 2013, and analyzed by R (Version 3.3.1 Patched for x64 Window system).
